# Postoperative Cyst Associated with Bone Morphogenetic Protein Use in Posterior and Transforaminal Lumbar Interbody Fusion Managed Conservatively: Report of Two Cases

**DOI:** 10.7759/cureus.485

**Published:** 2016-02-07

**Authors:** Eli M Baron, Diana M Mejía, Doniel Drazin, Neel Anand

**Affiliations:** 1 Neurosurgery, Cedars-Sinai Medical Center; 2 Department of Physical Medicine and Rehabilitation, University of Miami - Jackson Memorial Hospital; 3 Surgery, Cedars-Sinai Medical Center

**Keywords:** bone morphogenetic protein, spinal fusion, off-label

## Abstract

Bone morphogenetic protein use in spinal surgery for off-label indications continues to remain popular. One area where its use has known associated radicular complications is posterior or transforaminal lumbar interbody fusion. These complications include radiculitis, cyst development, and heterotopic ossification, amongst others. Typically, cyst development has been treated surgically. We present two cases of bone morphogenetic protein-related cysts treated medically and thus, present medical treatment as an alternative treatment option.

## Introduction

Bone morphogenetic protein (BMP) was initially identified by Urist in the early 1970s [1-3]. Since then, BMPs have been used for numerous clinical indications where new bone formation and fusion is required including craniofacial surgery, long bone fracture repair, and spinal surgery, among others [4-7].

Recombinant bone morphogenetic protein 2 (rhBMP-2), one of the transforming growth factors-β group, was approved by the FDA in 2002 for use in skeletally mature patients, in cages in anterior lumbar interbody fusion (ALIF) [8]. It promotes osteogenesis, helping mesenchymal cells differentiate into osteoblasts, and affects bone resorption [9-11]. The first human clinical trial reporting the success of rhBMP-2 in ALIF was Boden et al., in 2000 [12]. Since then, rhBMP-2 has become widely used as an alternative to autogenous bone grafting in lumbar anterior interbody fusion [13-15].

In addition to its FDA-approved use in the lumbar spine for ALIF (within a cage), its off-label use in the spine increased significantly in the last decade. Partially as a result of commercialization and high reported fusion rates, the use of rhBMP-2 in all fusions increased between 2002 and 2006 from 0.69% to 24.89% [16]. Using the Nationwide Inpatient Sample, Ong et al. noted the predominant use of spinal rhBMP-2 between 2003 and 2007 to be primary posterior lumbar interbody fusion (PLIF) or transforaminal lumbar interbody fusion (TLIF) (30.0%), followed by primary posterolateral spine fusion (20.4%) and primary ALIF (16.6%) [17]. Thus, off-label use of rhBMP-2 far exceeded its on-label use in ALIF cages during this time period in the United States.

Initial studies showed bone formation using BMP-2 to occur with equal or greater efficacy when compared with iliac crest autograft [18-19]. As increased use of the protein occurred, several authors reported significant morbidity related to its use [20-25].  RhBMP-2 has been related to numerous physiologic cascades responsible for both the benefits and the morbidity associated with its application. The protein induces cell proliferation, differentiation, and apoptosis, which are key elements in the osteogenesis process [26]. Conversely, it also promotes the expression of a number of inflammatory agents including interleukin-1a, 1b, 6, as well as tumor necrosis factor-a, which may be responsible for many of its side effects [11, 27]. In addition to inflammatory radiculitis, epidural heterotopic bone formation anterior to or in the spinal canal is also responsible for some of the complications related to rhBMP-2 in the disc space. Uncontrolled bone growth and cyst formation have been found compressing adjacent neural structures causing postoperative radiculopathy, leg pain, and motor and/or sensory deficits [20, 28-31]. These cases have been treated most commonly with surgical intervention aimed to decompress the affected neural structure.

We report two patients who developed a posterior cyst after the off-label use of rhBMP-2 in PLIF and TLIF surgery. Both patients presented with radiculopathy a few weeks to months after the surgery. The diagnosis was confirmed through postoperative MRIs that showed nerve root compression or presence of a postoperative cyst. These patients were treated conservatively, and no second surgical intervention was performed. After at least one year at follow-up, these patients showed complete fusion with calcification of the cyst walls and stabilization or resolution of the symptoms. These cases support a non-interventional treatment option for postoperative cyst formation after rhBMP-2 use during TLIF/PLIF. 

## Case presentation

### Case 1

**Figure 1:**
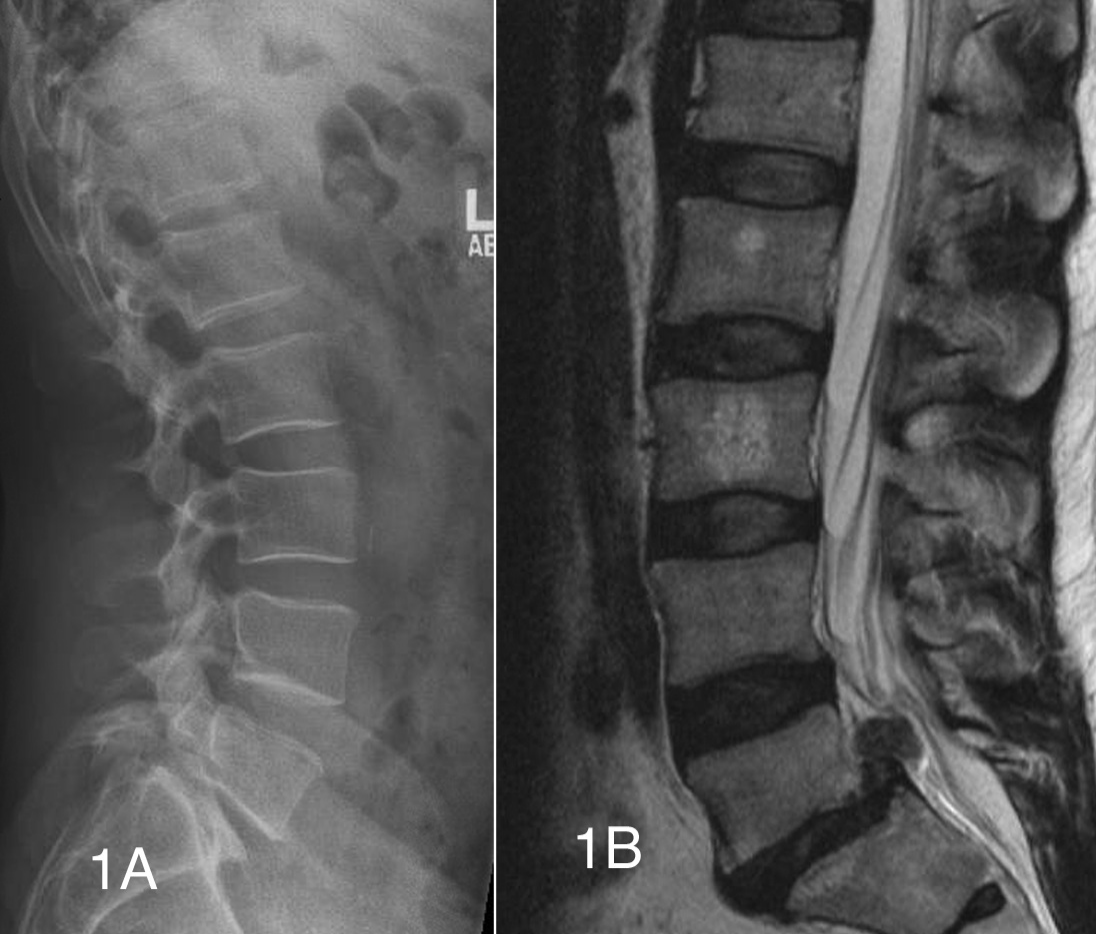
Preoperative lateral radiograph and t2 weighted sagittal MRI (Figure 1A) Lateral lumbar radiograph showing a grade I L5-S1 spondylolisthesis. (Figure 1B) T2 sagittal MRI reveals a large L5-S1 disc herniation in the setting of grade I L5-S1 spondylolisthesis.

A 48-year-old male presented with a history of two years of severe, worsening, right buttock and right posterior thigh pain. Six weeks prior to the visit and associated with the pain, the patient started to have right foot numbness in the second and third toe, which in the 4-5 days prior to the visit had extended to the bottom of the right foot compromising his walking. He also presented with occasional right calf numbness and spasms. Walking and standing exacerbated the lower extremity pain, while lying down in a fetal position provided some relief. The symptoms did not respond to conservative treatments which included analgesics and a lumbar support brace. The physical exam was normal except for numbness in the dorsum of the right foot and diminished right ankle reflex. Preop radiographs showed a Grade I L5-S1 lytic spondylolisthesis and MRI showed an L5-S1 disk herniation with clear protrusion into the spinal canal (Figures 1A, 1B).

The patient underwent L5-S1 radical discectomy and posterior lumbar interbody fusion (PLIF) with local bone autograft Grafton putty demineralized bone matrix and 3 mg of RhBMP-2 divided and placed into two polyetheretherketone (PEEK) spacers.

**Figure 2:**
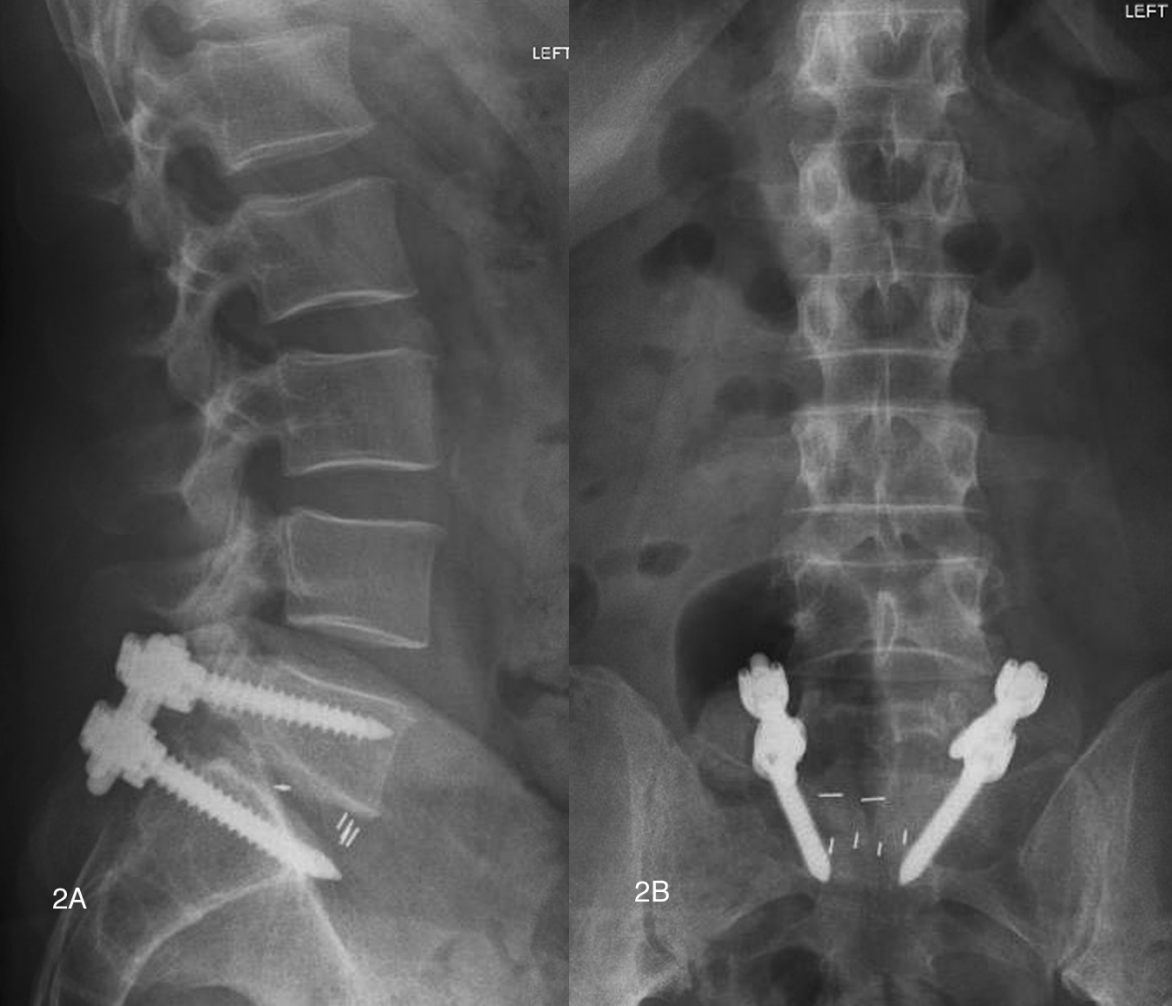
Postoperative lateral and AP radiographs (Figures 2A, 2A) Lateral and anteroposterior radiographs demonstrating appropriate PLIF graft and pedicle screw instrumentation placement.

After the surgery, the patient reported a significant decrease in the right lower extremity pain; however, the right foot numbness still persisted. One and a half weeks after the surgery, the patient started experiencing radiating aching pain through the right posterior thigh that stopped around the posterior right knee. He also had worsening hypersensitivity in his second and third right toes and tightness sensation from his right thigh all the way down the ipsilateral foot. Doppler ultrasound ruled out a possible deep venous thrombosis. X-rays of the lumbar spine demonstrated instrumentation to be intact with no lucency at the screw-bone interface (Figures 2A, 2B).

**Figure 3:**
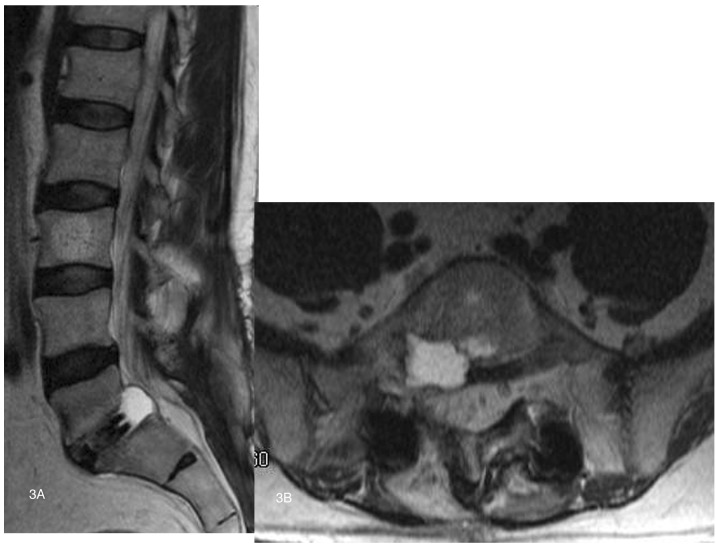
Postoperative T2 weighted sagittal and axial MRI imaging showing cyst (Figure 3A) T2 sagittal MRI. (Figure 3B) T2 axial MRI demonstrating hyperintense cyst emanating from the disc space and ventrally compressing the right L5-S1 lateral recess.

An EMG/NCV study of the lower extremities two months after the procedure suggested reinnervation of the right S1 nerve, but also showed a degree of acute denervation in his right L5 area. An MRI of the lumbar spine revealed a right-side fluid collection at L5-S1, visualized within the right neural foramen and lateral recess (Figures 3A, 3B).

Surgical decompression was offered to the patient; however he decided to defer the intervention and requested to be treated conservatively. The patient was treated with Neurontin 200mg twice daily and Mobic 7.5mg twice daily, which improved his symptoms.

**Figure 4:**
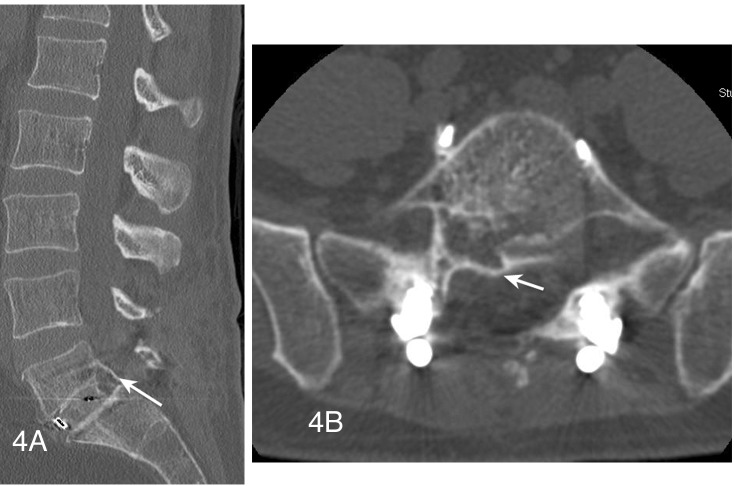
Postoperative sagittal and axial CT images showing calcification along former cyst wall (Figure 4A) Sagittal CT reconstruction image demonstrating a solid fusion across the disc space with calcification posteriorly along the previously seen cyst wall (arrow). (Figure 4B) This also visualized on an axial CT image (arrow) and appears to extend into an area of osteolysis along the posterior inferior vertebrae.

Fifteen months after the surgery, the patient had residual but stable right-side calf and foot numbness. However, the patient increased his activities and was satisfied with the results of the surgery. A one-year CT scan postop study showed a robust interbody L5-S1 level and light calcification along the borders of where the cyst was observed (Figures 4A, 4B).

### Case 2

A 29-year-old female with a history of left-sided L5-S1 microdiscectomy from several years previous developed a right-sided L5-S1 disk herniation. She presented with excruciating intractable back, buttock, and leg pain. There was evidence of a retrolisthesis at L5-S1, in addition to a collapsed disk at this level. The patient was also noted to have mild stenosis at L4-L5. After failing to improve from nine months of conservative care, surgery was offered.

**Figure 5:**
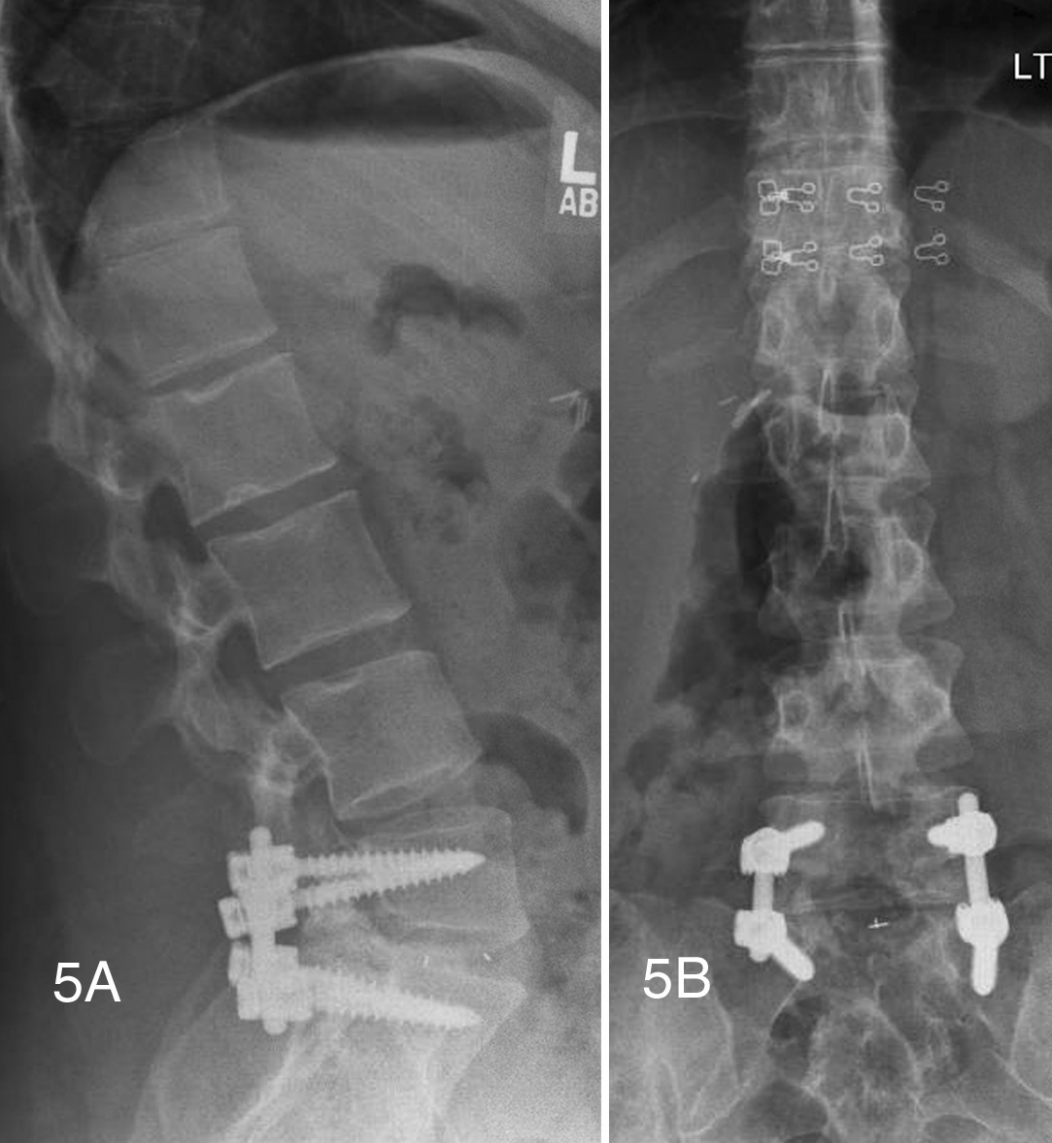
Postoperative lateral and AP radiographs (Figures 5A, 5B) Lateral and AP radiographs showing proper pedicle screw and oblique PLIF cage placement using TLIF technique.

The patient underwent right-sided L5-S1 transforaminal lumbar interbody fusion in addition to L5-S1 laminectomy and a decompressive laminotomy at L4-L5. Bone morphogenetic protein was used in the intervertebral cage and for a contralateral posterolateral facet fusion. An oblique PLIF cage was inserted via TLIF technique containing 2.1 mg of rhBMP-2 (Figures 5A, 5B).

**Figure 6:**
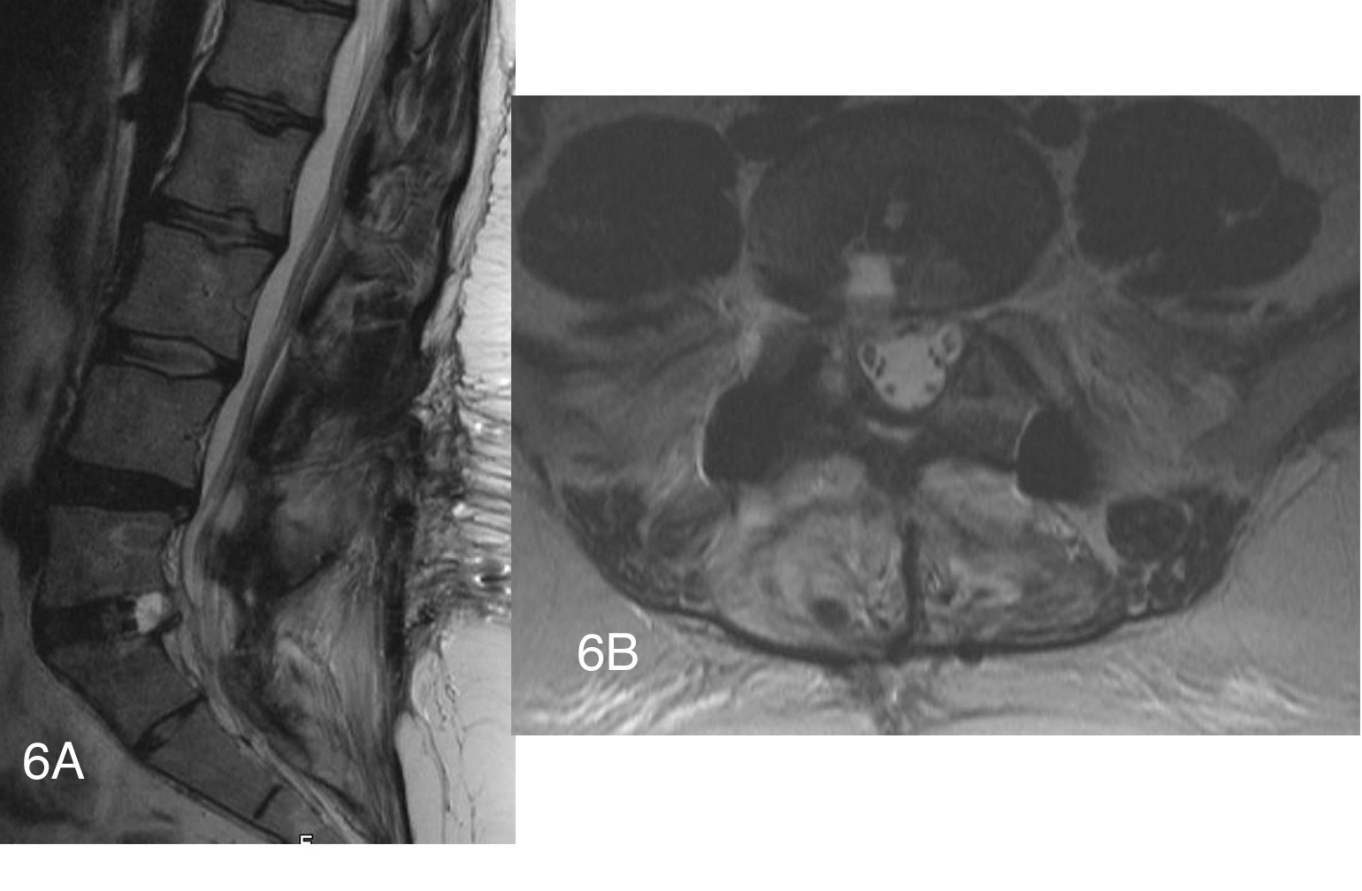
Postoperative T2 weighted sagittal and axial MRI imaging showing cyst T2 weighted sagittal and axial MRI imaging demonstrating right sided cystic  flud collection in the disc space. The right S1 nerve root appears to be displaced posteriorly on the sagittal image.

Several months after surgery, the patient was noted to have new onset buttock pain and right leg pain. T2 MRI of the lumbar spine revealed a hyperintense fluid collection extending just posterior to the disc space on the right side. On sagittal imaging this could be seen to be displacing the right S1 nerve root posteriorly (Figures 6A, 6B).

**Figure 7:**
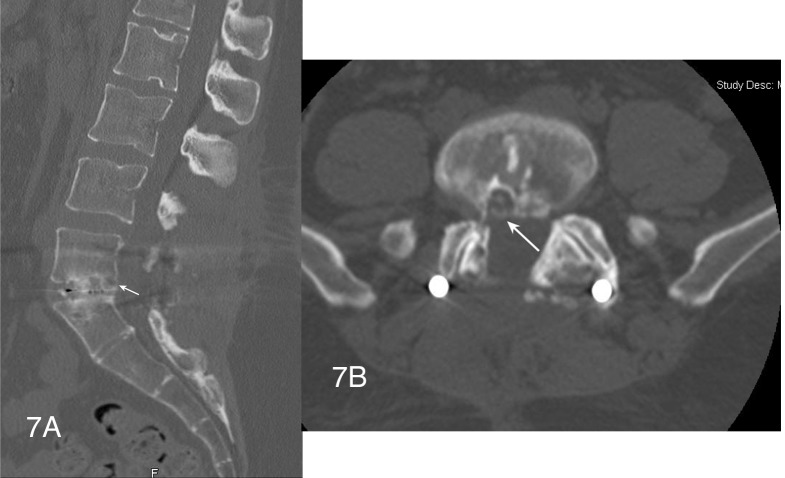
Sagittal and axial postoperative CT images showing calcification along previously noted cyst wall (Figures 7A, 7B) Sagittal and axial postop CT images at 1 year postop displaying a solid interbody fusion. The posterior aspect of where the cyst wall was (arrows) is noted to be calcified.

Inflammatory markers, including ESR and CRP, were obtained and found to be within normal limits. Given this, a right L5-S1 transforaminal epidural steroid injection was performed with good relief of symptoms; over several months her leg pain resolved. Follow-up CT scan at one-year post-op displayed a solid interbody fusion and solid fusion of the left L5-S1 facet joint (Figures 7A, 7B).

## Discussion

In the past decade, the use of rhBMP-2 has become increasingly common to promote lumbar interbody fusion. After the FDA approved the use of rhBMP-2 in the LT cage for ALIF, off-label use of BMP increased significantly, specifically in TLIF/PLIF and posterolateral fusion [16-17]. Significant radicular complications, however, have been reported associated with its use, both in the early and late perioperative period [20, 22, 24, 30, 32-36].

Radiculopathy after TLIF/PLIF has been reported at 0-18% in studies where BMP was used [21]. In a study conducted by Rihn et al., 14% of the patients presented with ipsilateral, postoperative leg pain in a dermatomal distribution after TLIF with BMP (3% without BMP) [24]. Conversely, only 16% of the cases were related to heterotopic bone formation. Causes of radiculopathy can be divided into two categories: compressive and non-compressive. Stenosis, heterotopic ossification (HO), fluid collection, and scars are examples of causes of compressive radicular pain. Non-compressive radiculopathy causes include inflammation of the nerve root (usually without imaging findings and seen most commonly) and infection [26]. Mindea et al., reported that 11% of the patients who underwent TLIF with rhBMP-2 had radicular symptoms with no abnormality on postoperative CT scans [34]. In a retrospective study of 199 patients who underwent single level TLIF, 86 patients received rhBMP-2 [24]. Of these, a hydrogel sealant (Duraseal; Confluent Surgical Inc., Waltham, MA, USA) was used in 37 patients. The Duraseal group showed a 5.4% incidence of postoperative radicular symptoms versus 20.4% when the hydrogel sealant was not used (with rhBMP-2). Duraseal is believed to provide a protective layer for nerve roots and the dural, effectively isolating them from the rhBMP-2. Nevertheless, concerns about postoperative swelling associated with use of Duraseal, and subsequent radicular or thecal sac compression, have been raised which may temper enthusiasm about its use in this setting [34, 37]. Others have considered the use of fibrin glue for this purpose [38].

Rates of HO after TLIF/PLIF where rhBMP-2 has been used also vary widely. Haid et al., in a prospective, randomized, non-blinded, two-year study at 14 investigational sites comparing PLIF with rhBMP-2 to autogenous iliac bone graft reported on HO as noted on CT, and found 75% of the patients who underwent PLIF with rhBMP-2 had HO compared to 12.9% of cases in subjects where BMP was not used [39]. In an observational study with prospective CT analysis, Joseph and Rampersaud noted an incidence of epidural bone formation of 20.8% in patients undergoing PLIF or TLIF with the use of rhBMP-2 compared with an incidence of 8.3% in patients undergoing PLIF/TLIF with local autogenous bone graft [29]. Rates of HO reported in numerous series vary from 0 -75%, with many asymptomatic cases [21]. The mechanism of this HO formation after TLIF is not fully understood but may involve a chondrocyte differentiation pathway [40].

Cyst formation is a less commonly reported complication related to the off-label use of rhBMP-2. Just a few case reports in the literature discuss the presence of cyst formation associated with radicular pain. Choudhry et al., described the presence of a symptomatic inflammatory cyst presenting six weeks after TLIF [20] wherein 8.4 mg of rhBMP-2 was used inside a PLIF cage inserted using oblique TLIF technique [41].  The authors reported the case of a patient who developed inflammatory fibroblastic cyst formation around the rhBMP-2 sponge, which compressed the L4 nerve root at its axilla. Decompression and excision of the cyst were performed in a second surgery eight weeks after the index procedure with resolution of patient’s symptoms. Histopathologic specimen analysis confirmed the presence of a rhBMP-2 sponge material surrounded by mild, chronic inflammation where hemosiderin-laden macrophages with foci of calcifications were noted.

Than et al., described a case of back and right lower extremity pain 20 months after initially successful L4-L5 fusion for spondylolisthesis [42] wherein 2.1 mg of rhBMP-2 was used in the disc space. Postoperative MRI showed a right-side cystic mass at L4-L5. Peripheral calcification was demonstrated on CT scan. Surgical resection of the mass was performed in this patient with improvement in his symptoms. Histopathology of the resected tissue revealed the presence of new connective tissue as well as new bone and cartilage formation.

Mannion et al., reported on 30 patients undergoing 36 spinal levels of TLIF and PLIF with low-dose rhBMP-2 where 1.4 mg was used per level fused [23].  They followed all patients clinically and obtained CT scans six months out from surgery. They note two patients with inflammatory cysts and two patients with HO. Of the two patients with cysts, one had severe symptoms that resolved spontaneously and continues to be followed clinically. The other patient had their cage retropulsed into the disc space; that patient underwent cyst drainage and cage reinsertion with resolution of the patient’s radicular symptoms.

Surgical exploration with cyst drainage/resection, the most common treatment for cyst formation after lumbar interbody fusion with rhBMP-2, has been reported to provide immediate symptom relief and thus has been advocated [20, 42].  Another advantage of re-exploration is the direct visualization of the affected area, which can be especially beneficial in the presence of compressive scar tissue, seroma, or hematoma. The disadvantage, however, is that patients have to undergo another surgical intervention that also carries its own risks and complications including infection, durotomy, and possible neurologic injury. The first author (E.B) had a case where cyst drainage resulted in marked weakness of a patient’s dorsiflexors where at surgery there was no visualization of a frank root injury. Post-op MRI showed excellent cyst removal, and CT myelography failed to reveal any abnormalities. A second intervention in the same area also increases the risk of scar tissue formation that can subsequently compress the surrounding neural structures as well as irritation of the nerve root.

Both patients in this case report exhibited total recovery from their initial symptoms after their single level TLIF or PLIF. RhBMP-2 was used in both cases with solid interbody fusion in both patients. Several weeks after their fusion procedures, however, the patients’ radicular pain reappeared. In both cases, MRI of the lumbar spine confirmed adequate position of the cages and the hardware material. Instead, cyst formation was observed at the level of the fusion. Compression of the adjacent nerve roots or local inflammation was found to be the cause of their postoperative symptoms. Treatment options were discussed with the patients, and they both declined a possible surgical excision of the cyst. Both patients received conservative treatment which included oral analgesics, and an epidural injection in one of the cases. Partial relief of the symptoms was observed in both patients. Follow-up images showed the cysts to be stable in size in addition to peripheral calcification observed at the twelve-month follow-up. This finding further supports rhBMP-2 as the cause of these inflammatory cyst formations.

Measures to prevent inflammatory cyst formation as well as HO may be taken in order to avoid some of the morbidity related to the procedure. Using lower doses of rhBMP-2 may achieve successful fusion and avoid some of its inflammatory side effects [23, 43]. Excessive, uncontrolled bleeding could predispose migration of rhBMP-2 to the spinal canal and may itself be a risk factor for HO [44]. Effective hemostasis theoretically could decrease the incidence of HO and postop cyst formation. Irrigation of the surgical field after cage insertion with rhBMP-2 may also reduce inflammation in the spinal canal by washing away any unbound rhBMP-2 [34]. Additional investigation on Duraseal and other barriers as a prophylactic measure should aim to determine their safety and efficacy to decrease postoperative radiculopathy related to rhBMP-2 . Finally, rhBMP-2 should be placed in cages as far away from the spinal canal as possible. When performing TLIF, we prefer a C-shaped graft positioned maximally anteriorly with the rhBMP-2 in the cage to minimize the risks of its effect on the canal; additionally, this technique has the benefit of increased lordosis over other TLIF techniques [45].

## Conclusions

Cyst formation related to rhBMP-2 use in PLIF and TLIF has been reported. Cysts may be compressive causing neurologic symptoms. Successful management options include both nonoperative measures and surgical management. An emphasis should be placed on prevention through judicious use of low-dose rhBMP-2, proper cage placement containing the protein, hemostasis, appropriate post cage insertion irrigation, and possibly the use of barrier materials.
